# RNA Editors, Cofactors, and mRNA Targets: An Overview of the C-to-U RNA Editing Machinery and Its Implication in Human Disease

**DOI:** 10.3390/genes10010013

**Published:** 2018-12-27

**Authors:** Taga Lerner, F. Nina Papavasiliou, Riccardo Pecori

**Affiliations:** 1 Division of Immune Diversity, Program in Cancer Immunology, German Cancer Research Centre, 69120 Heidelberg, Germany; t.lerner@dkfz-heidelberg.de; 2 Division of Biosciences, Uni Heidelberg, 69120 Heidelberg, Germany

**Keywords:** RNA editing, epitranscriptome, neurological disorders, AID/APOBEC

## Abstract

One of the most prevalent epitranscriptomic modifications is RNA editing. In higher eukaryotes, RNA editing is catalyzed by one of two classes of deaminases: ADAR family enzymes that catalyze A-to-I (read as G) editing, and AID/APOBEC family enzymes that catalyze C-to-U. ADAR-catalyzed deamination has been studied extensively. Here we focus on AID/APOBEC-catalyzed editing, and review the emergent knowledge regarding C-to-U editing consequences in the context of human disease.

## 1. Introduction

Epitranscriptomics is a recently coined term that refers to the study of RNA modifications and their effects on the transcriptome. Included under this umbrella is everything from RNA tertiary structure, to processing, stability, localization, and translational efficiency. To date, more than 150 distinct types of RNA modifications have been identified, primarily in ribosomal RNA (rRNA) and transfer RNA (tRNA) [1]. Many of these are suspected to be essential, as loss of tRNA or rRNA modification “writers” or “readers” are often found to be associated with diseases (e.g., cancer—[2])

A small subset of these modifications have also been found in messenger RNA (mRNA) (reviewed in [3]), where they are thought to represent a new mode of regulation of the biological information influencing the fate of each transcript. The most prominent of these modifications is the deamination of adenosine or cytosine (to inosine (decoded as guanosine) or uracil, respectively). Because these modifications effectively lead to single- nucleotide changes in mRNA, the phenomenon has also been termed “RNA editing”. This is distinct from the “editing” described in the mitochondrion of trypanosomes, which refers to the insertion or deletion of uridines to correct the “frame” [4].

mRNA deamination is mediated by two classes of enzymes: the “adenosine deaminases that act on RNA” (ADARs) and the cytidine deaminase family of “Apolipoprotein B mRNA editing enzyme, catalytic polypeptide-like” (also known as the activation-induced deaminase AID/APOBEC family). Here we will focus on AID/APOBEC-mediated RNA editing, and review the impacts of such editing on single transcripts, the cell, and the whole organism.

## 2. AID/APOBECs: A Common Path through Evolution

APOBEC1 was the first member of the AID/APOBEC family to be discovered and successfully cloned [5]. This enzyme deaminates a specific cytosine in the long apolipoprotein B (*Apob*) pre-mRNA; the C-to-U editing event recodes a CAA codon (Q2180) to a stop codon, resulting in a truncated form of the APOB protein, called APOB-48 [5,6]. Because of this functionality, the *Apob* RNA editor was termed “apolipoprotein-B mRNA editing enzyme, catalytic polypeptide-1” (APOBEC1). The rest of the family derives its name from this founding member.

There are several members in the AID/APOBEC family, and all of them (except for APOBEC2 and APOBEC4) have C-to-U deaminase activity on single-stranded DNA or RNA. All family members share the structural and catalytic backbone of the zinc-dependent deaminase superfamily, which also comprises proteins such as ADATs and ADARs, which are involved in the metabolism of purines or pyrimidines. They are also evolutionarily conserved. The first member of the family to appear in the vertebrate lineage is the activation-induced cytidine deaminase (AID), a molecule that is crucial to antibody diversification [7], whose appearance coincides in sharks with the dawn of adaptive immunity [8]. APOBEC1 appeared soon thereafter, from an inverted duplication of the *Aid* locus [9]. In primates, *Aid* and *Apobec1* are linked, with the *Apobec1* locus being only 1 Mb away from the *Aid* locus [9]. The main difference between AID and APOBEC1 is the primary physiological choice of the nucleic acid substrate; single stranded DNA for AID, and RNA for APOBEC1 [10,11]. A secondary difference is the presence of a longer coding sequence at the 3′ end of the *Apobec1* gene, which might have implications for dimerization [12,13], nucleocytoplasmic transport, and possibly substrate binding [14,15].

The *Apobec3* (A3) locus [16], appeared after the divergence of the marsupial and placental lineages. A duplication formed the first two A3 genes from which all the other A3s have evolved [9]. In some species, for example in rodents, these two original genes fused into a single gene with a double zinc-coordinating domain.

## 3. APOBECs as RNA Editors

AID/APOBECs show physiological preference for specific nucleic acid substrates (e.g., RNA or DNA) but most of them can deaminate both, under certain conditions [15]. Here we will focus on the enzymes for which RNA editing activity has been reported, i.e., in APOBEC1, A3A [17], and A3G [18]). We will first review how these molecules were discovered, whether they might require co-factors for activity, and will discuss their known mRNA targets. Finally, we will consider emergent knowledge on how mutations within the editing enzymes or their co-factors (or their mRNA targets) might mediate human disease.

## 4. APOBEC1

### 4.1. Key Facts and Controversies

The full length APOB protein (APOB-100) in humans is synthesized in liver, and is the major protein component of plasma low-density lipoproteins (LDLs). In contrast, the edited form (APOB-48) is produced in the small intestine, and is essential for the secretion of chylomicrons [19,20]. Lipoproteins containing APOB-48 are quickly cleared from plasma, and are not converted into LDL. Mammals that edit *Apob* in liver (horses, rats, mice and dogs) have low levels of plasma LDLs [21]. As soon as APOBEC1 was defined as the key enzyme in *Apob* editing, the Innerarity group overexpressed APOBEC1 in the liver of mice and rabbits in an attempt to lower plasma LDL and demonstrate therapeutic potential. Instead, they uncovered an unexpected phenotype: these animals developed hepatocellular carcinoma [22]. This was the first association between APOBEC1 editing activity and a specific disease.

Shortly after the discovery of APOBEC1, in vivo experiments showed that RNA editing by APOBEC1 is not redundant; APOBEC1 is the only responsible protein for this deaminase activity [23,24]. Loss of APOBEC1 resulted in viable mice, despite alterations in lipoprotein metabolism [25]. These initial experiments also showed that APOBEC1 was essential for editing, but that additional factors were also important. In vivo biochemical work showed that APOBEC1 alone in the presence of *Apob* RNA was unable to catalyze editing [5,26]. Indeed, the RNA binding affinity of APOBEC1 is rather low [27]. A few years later, two groups showed that the addition of an RNA-binding protein, termed the APOBEC1 complementation factor or A1CF (ACF), was sufficient in vitro to complement efficient C-to-U editing in the presence of APOBEC1 and *Apob* mRNA [28,29], thus defining a minimal editing complex.

From there and for the next 15 years, conflicting information was gathered regarding APOBEC1. *Apob* editing is a nuclear event [30,31], and several papers demonstrated the need for nucleo-cytoplasmic transport. However, the mechanism was debated. Several investigators maintained that APOBEC1 contains an NLS in its N-terminus, and an NES in the C-terminus [5,32,33,34], and that APOBEC1 was necessary and sufficient to shuffle the complex between the cytoplasm and the nucleus [32,33]. However, ACF also contains an NLS [35]. The question became one of dependencies. Some investigators noted that APOBEC1 import/export is importin-α- and exportin-1-dependent respectively, and inhibited by leptomycin B—thus suggesting that the shuttling was APOBEC1-dependent [32,36]. Others suggested that ACF shuttling was dominant, and a transcription-dependent process that is inhibited by actinomycin D treatment [35].

Another dispute regarded the function of the C-terminal part of APOBEC1. This portion of the protein distinguishes APOBEC1 from the rest of the family, and is probably important for homodimerization [12], which in turn was thought to be important for editing activity. C terminally deleted mutants of APOBEC1 cannot dimerize, and are deficient in RNA editing activity [37]. APOBEC1 C terminal domain-interacting proteins were mostly inhibitory to editing [13,38,39,40,41,42,43]. Nevertheless, APOBEC1 with partially deleted C-terminal domains are still able to edit RNA in vitro, indicating that the relation between editing and dimer formation is still not clear [44,45]. It is not a surprise that these data that rely on experiments performed in different cells lines, often overexpressing tagged proteins from different species, resulted in conclusions that were not always in agreement with each other. To date, there is no physiological cell line model for APOBEC1. In addition, good antibodies for APOBEC1 do not exist, and thus the ability to detect physiological levels of the protein in its proper context is missing. Such tools will be necessary to clarify how the APOBEC1 cofactor–RNA complex works.

### 4.2. Cofactors

Before the discovery of ACF, genetic experiments determined several sequence elements on *Apob* mRNA that were required for efficient editing by APOBEC1 [46,47]. These sequence elements were found within 50 bp surrounding the target C. Two elements were described: an element 5′ to the edited C, and a 3′ (11 nt) element, termed the mooring sequence. These local sequence elements also benefit from a surrounding region that was AU rich [48,49]. Based on this information, several papers hypothesized the possibility that these elements were important for the creation and stabilization of a secondary RNA structure, amenable to APOBEC1 editing [50,51,52].

The hypothesized minimal editing complex, consisting of ACF, APOBEC1, and *Apob* mRNA, led to a model in which ACF served as the RNA-binding protein (RBP), binding the mooring sequence [48,53] and recruiting (or positioning) APOBEC1 to the editing site [36,54,55]. Work from several labs suggested that the complex itself was co-ordinately regulated. Both APOBEC1 and ACF have splice variants that modulate expression [56,57,58,59], and both can be posttranscriptionally modified (which impacts their subcellular localization, editosome assembly, and editing activity) [60,61,62].

The model of an APOBEC1-ACF editosome was upended with the discovery of another RBP, the RNA-binding motif protein 47 (RBM47), as a cofactor indispensable for *Apob* editing in vivo, and an effective substitute for ACF in vitro [63]. Although this was not entirely unexpected [42], it was further confirmed by a new *Acf*^−/−^ mouse model in which a different gene targeting strategy resulted in viable mice [64] (in contrast to the original knockout that suffered from early embryonic lethality [65]). In this model, the loss of ACF did not result in a loss of editing in the liver or small intestine, either in *Apob*, or in additional known edited transcripts [66,67]. This helped to explained a finding from the original knockout paper that reported that heterozygous *Acf*^+/−^ mice were viable, but did not show a decrease, but rather an increase in intestinal *Apob* editing [68]. A very recent paper resolves this conundrum by using genetic means to demonstrate that ACF and RBM47 interact with (and attract APOBEC1 to) different sets of transcripts [69]. Specifically, these authors showed that a loss of ACF or RMB47 results in the loss of non-overlapping sets of transcripts (as assessed by transcriptome sequencing). Moreover, the loss of both ACF and RBM47 does not completely ablate APOBEC1-mediated editing. This suggested that APOBEC1 is able to induce RNA editing alone under physiological settings, or that additional RBP cofactors exist (perhaps expressed in a tissue or stage specific manner) that recruit APOBEC1 to specific transcripts. APOBEC1 is able to edit mRNA on its own in vitro ([70,71]) and in vivo (under overexpression or ectopic expression conditions a phenomenon described as “hyper editing” [22,32]). Whether this editing can happen under normal conditions remains unclear. The alternative possible model suggests that ACF and RBM47 (and perhaps other RBPs) exhibit distinctive tissue- and transcript-specific functions that modulate APOBEC1-dependent RNA editing site selection and activity [69].

This possibility is further supported by the discovery of another putative paralogue of ACF, DND1, which seems to interact with APOBEC1 in vitro though only weakly in 293T transfected cells [72]. *Dnd1^ter^* mutant mice develop testicular germ cell tumors (TGCT) [73], whose incidence can be modulated by the dosage of an *Apobec1*-null allele [74]. In this context, *Apobec1* acts as a TGCT enhancer, an effect that is inherited in a conventional manner in the paternal germ-lineage, and is also transgenerationally inherited for at least two subsequent generations (regardless of *Apobec1* genotype). These results suggest that a maternally inherited *Apobec1* mutation can affect the tumorigenic susceptibility of two generations of offspring in a transgenerational epitranscriptomic manner [74]. The possibility that DND1 is another ACF/RBM47-like cofactor for APOBEC1 had previously been suggested [75]. Future studies will be necessary to confirm the interaction between APOBEC1 and DND1, and to understand which part of APOBEC1 interacts with RBM47 (as has been done for ACF [54]). Finally, it will be important to identify which transcripts are recruited by the distinct RRM motifs of these cofactors (perhaps in a sequence specific manner [76]). In this context, it is interesting to note that DND1 can inhibit miRNA-mediated RNA decay [77], and that APOBEC3G is able to modulate this function of DND1 [78], suggesting binding on similar transcripts/motifs of these two proteins.

### 4.3. RNA Targets and Their Functional Relevance

The best characterized RNA target of APOBEC1 is *Apob*, whose editing is essential for proper lipoprotein metabolism. The absence of editing leads to important lipid metabolic phenotypes in *Apobec-1^−/−^*. These mice express *apoB100*-only, and show spontaneous hypercholesterolemia when crossed into the *LDLR^−/−^* background [79]. Finally *Apobec-1^−/−^* mice crossed into a conditional intestinal *Mttp* deletor background result in lethal intestinal lipotoxicity [80].

APOBEC1 was initially thought to only edit *Apob*, and to be expressed only in APOB-expressing tissue (the small intestine in humans and the small intestine and liver in mice [24]). When the dependencies of APOBEC1 editing on the mooring sequence were discovered, other target RNAs were proposed. Neurofibromatosis type 1 (*Nf1*) mRNA was found to be edited within an alternatively spliced exon in a subset of patients with peripheral nerve sheath tumors [81,82]. *Nat1* mRNA was found to be edited in hepatocellular carcinomas, in positions with surrounding sequence homology to the mooring sequence [83].

More recently, it has become clear that APOBEC1 is expressed by most murine immune cell types [67,84,85], where it targets mostly the 3′ UTRs of tens, if not hundreds of transcripts [66,67]. *Apobec-1^−/−^* mice show a neurological dysfunction characterized by a proinflammatory phenotype [86]. In this context microglia are more ‘activated’, and C-to-U editing was shown to be relevant in lysosomal-associated protein-2 (LAMP-2) protein production [86]. This suggests that APOBEC1 RNA editing may play an important role in maintaining brain homeostasis [87]. C-to-U editing was also found to create a supramedullary glycine receptor (GlyRs) isoform, GlyRα3^P185L^ [88]. Neurons do not express *Acf* or *Rbm47*, again suggesting the possibility of additional co-factors that are involved in editing [86,89]. Lamp1 and related molecules were shown to be edited in murine macrophages (a myeloid population related to microglia) [86]. C-to-U editing is much more widespread than previously thought. Editing is almost always restricted to 3′ UTRs (with a few exceptions), suggesting functions in the regulation of mRNA stability, localization, translation [66,67]. In addition, because C-to-U editing occurs at the same time as splicing and poly-adenylation [30], and also as APOBEC1 prefers to bind to AU-rich regions [90], the intriguing possibility arises that editing can generate polyadenylation signals in targeted transcripts by generating AAUAAA motifs [91].

Recent work has demonstrated that APOBEC1 editing can create sequence variability within a population, generating different subsets of cells with a distinct complement of edited transcripts [84], something that has also been proposed for ADAR-mediated editing [92]. Additionally, it has also been suggested that APOBEC1 editing on different transcripts belonging to the same pathway can be associated with specific cellular functions [85]. This requires further study, potentially by using targeted editing tools on an APOBEC-deficient background. Such tools are already available for A-to-I editing, reviewed by [93], but not for C-to-U. They will be absolutely necessary for a complete understanding of the role of C-to-U editing on single transcripts, pathways, and cells belonging to different tissues.

### 4.4. Additional Functions

APOBEC1 prefers binding to AU-rich regions common in 3′ UTRs. It has been proposed that the binding of APOBEC1 to the 3′ UTRs of transcripts such as *c-Myc*, *Tnf-α*, and *Il-2*, which normally exhibit rapid turnover, is sufficient to result in increased mRNA stability in vitro [90] and in vivo [94], even in the absence of editing. Similarly, APOBEC1 can bind and stabilize the cyclooxygenase 2 (COX-2) mRNA in mouse enterocytes, resulting in an increase in prostaglandin E2 (PGE2) synthesis [95]. This effect correlates to the inability of *Apobec1*^−/−^ mice to properly regenerate small intestinal crypts after radiation injury (which is COX-2 and PGE2 dependent) [95]. Increased PGE2 production was also associated with colon tumor development and progression [96,97]. This matches experimental data where *Apobec1* deficiency, in mice genetically predisposed to developed intestinal polyposis (*Apc^min/+^*), results in increased apoptosis and reduced proliferation and tumor burden [94]. Editing was not exhaustively examined, in any of those papers (e.g., by transcriptome or even amplicon deep sequencing). However, these findings suggest that APOBEC1 can scan regions of transcripts that are AU-rich, and either do not contain substantial amounts of C (and are therefore not “editable”), or do not contain those in an editable conformation. Mutants that distinguish APOBEC1 binding vs editing in vivo do not exist (some biochemical findings are available [98]). Evidence of separate binding vs catalysis functions must be gathered with the help of specific function mutants, and the generation of mice carrying Apobec1 alleles with mutations that abolish binding vs catalysis.

APOBEC1 is able to deaminate single strand DNA (ssDNA). This mutagenic activity is cofactor-independent and significantly higher than AID [10,99]. APOBEC1 DNA editing activity was also shown on herpes and hepatitis viral genomes [100,101] and in mammalian cells [11]. In this context, APOBEC1 somatic mutations have a mutational signature which is present in esophageal adenocarcinomas [11]. In addition both AID and APOBEC1 were reported to have 5meC deaminase activity, suggesting a new role as DNA modifiers in cell reprogramming [102].

The question of how cells control APOBEC1 substrate selection (RNA over ssDNA) mitigating the oncogenic potential of APOBEC1 has never been formally addressed. However, it is interesting to note that mutations of RBM47, are linked with carcinogenesis, and it is a potential tumor suppressor gene. Therefore is tempting to speculate that cofactors play an essential role in controlling APOBEC1 substrate selection.

## 5. APOBEC3

### 5.1. Key FACTS and Controversies

The A3 family members of the AID/APOBEC family are believed to be a result of a process of gene duplications of the AID/APOBEC1 locus, or the APOBEC2 locus and then divergence and evolution of the genes over millions of years [9]. Humans and non-human primates have the highest number of members of the A3 family, seven (A3A, A3B, A3C, A3D, A3F, A3G, and A3H). Other mammals have fewer (e.g., cats and cattle have three members, whereas mice have only one) [103]. The best-studied functions of the A3s are in innate immune responses to retroviruses and endogenous retroelements, although other types of viruses can also be inhibited by these proteins [104,105,106,107,108,109,110,111,112,113,114]. The burden of retroelements is believed to be responsible for the expansion of A3s from one member in mice, to seven in humans, leading to fewer active retroelements in humans, compared to mice [115,116]. Polymorphisms in human A3s can result in variations in the antiviral/retroviral activity of these enzymes affecting a person’s susceptibility to certain viruses and associated co-morbidities, such as HBC-associated hepatocellular carcinoma [117,118].

The antiviral activity of the A3s was originally defined for A3G; it inhibited HIV in the absence of its viral neutralization partner Vif [119]. After its discovery, A3G created some controversy with conflicting data being published. There was some debate about the relation of the antiviral activity with reverse transcription; some groups claimed that the activity was independent of reverse transcription [120], while others claimed that A3G interacts with and inhibits reverse transcription [121]. The current general consensus is that deamination of the viral genome occurs at the same time as reverse transcription. Other controversies arise, surrounding the packaging of A3G into virons. The importance of A3G packaging and anti-viral activity was not debated but the mechanism and elements involved were debated. Different groups argued that the mechanism for packaging is RNA-independent, and mediated only by an interaction with viral Gag [122,123] versus those arguing that packaging is RNA-dependent [124,125]. The mechanism of the packaging of A3G remains unclear to date.

The prevailing model is that all A3s are more or less associated with the deamination of deoxycytidine to deoxyuridine in ssDNA formed during the viral/retroelement life cycle. This process leads to G to A hypermutations in the viral genome, ultimately reducing viral fitness [126]. It is not entirely clear how the A3s specifically identify their targets, and there are a few insights on how cellular DNA is generally avoided. Most of the A3s (A3G, A3D, A3F, and A3H haplotype II) do not come in contact with cellular DNA, and are maintained in the cytoplasm [127]. A3G binds RNA, and this is believed to be part of the mechanism that maintains its location in the cytoplasm (but not the only mechanism, as it is retained in the cytoplasm even without RNA binding) and inhibits its hypermutation of ssDNA [127,128].

However, not all A3 family members are restricted to the cytoplasm. Specifically, the A3H haplotype II which is cytoplasmic also has RNA binding capabilities, whereas haplotypes I, II, and VI which are all nuclear do not bind RNA [129]. As well, A3C can be found both in the nucleus and in the cytoplasm. A3A is mostly expressed in monocytes/macrophages, and under normal circumstances, it is cytoplasmic and therefore not genotoxic. However, when it is expressed/overexpressed in other cell types, it can also be found in the nucleus [130]. This suggests that some factor in the monocytes/macrophages that is generally absent in other cells is actively maintaining A3A in the cytoplasm. The human Tribbles 3 protein, a pseudokinase, could be such a candidate, as it interacts with both A3A and A3C and was found to decrease A3A editing of nuclear DNA in a concentration-dependent manner, thus protecting genetic integrity [131]. Finally, A3B has a strong NLS, resulting in only nuclear expression [132]. A3B genotoxicity seems to be mitigated under normal conditions by very low expression, although what exactly regulates its expression is yet unknown.

### 5.2. Additional Functions and Disease Associations

#### 5.2.1. DNA Mutation (Kataegis)

Despite careful control of A3 expression and localization, sometimes misregulation occurs, and this is associated with cancer. In particular, A3B and A3A are found to be overexpressed in some cancers and/or are associated with kataegis mutation (literally describing “rainfalls” of C-to-T mutation, particularly at promoter regions [133]. This has since been termed mutational signature 2 and 13 in these cancers [134,135,136,137,138,139,140,141,142]. A polymorphism where A3B is deleted and its 3′UTR replaces a deleted A3A 3′UTR results in higher levels of A3A and an increase in cancer risk in Asian populations [143]. It is unknown whether these genes are cancer drivers or a downstream consequence of other cancer driver genes. They do contribute to genetic instability and heterogeneity with important consequences to prognosis (which can be both positive and negative, depending on the cancer type) and cancer therapy [144,145,146]. A3G has also been associated with a negative prognosis in colon carcinoma patients with hepatic metastasis [147,148] and in T cells, it was associated with increased tumor infiltration and a positive prognosis [149]. It is also a target for cancer therapy, as it enhances tumor resistance to radiotherapy [150,151]. The mechanism of action of A3G in cancer remains unclear, and the mechanism appears to be unrelated to DNA deamination; however, in the case of resistance to radiotherapy, it is believed to promote DNA repair in a deaminase-dependent manner [147,148,149,150,151].

#### 5.2.2. RNA Editing

Traditionally, the A3s were considered to work only on ssDNA, and to bind RNA in a non-specific and non-selective manner [152]. However, in 2015, an RNA editing function for A3A was described in human monocytes/macrophages [17]. Editing levels were affected by hypoxia and the polarization of the macrophages [17] and transient ectopic overexpression of A3A in HEK293Ts also resulted in RNA editing of a large number of genes [153]. Later the same group went on to describe the RNA editing capabilities for A3G, again in the HEK293T cell system [18]. They also suggest that there is an increase in RNA editing as a response to mitochondrial stress in NK cells and lymphocytes [154].

Whereas most of the work on A3s and RNA editing involved overexpression, it remains clear that many of the A3s do bind RNA (reviewed in [155]). C-to-U editing in 3′ UTRs is readily apparent in transcriptome data from human cells or tissues (e.g., macrophages and others), where APOBEC1 is not expressed. A3A has been found to be associated with the editing of similar sets of transcripts as APOBEC1 in mouse macrophages [85], an activity that was upregulated upon interferon stimulation [156]. Thus, while the relevance of editing remains unclear in the context of the A3 family, it remains largely unexamined. Since APOBEC1 is absent in most human tissues, including macrophages and other immune cells, it is possible that A3A (and perhaps additional family members) have adapted and evolved to carry out similar roles in humans. In that regard, it would be interesting to assess the known SNPs and polymorphisms in A3s (especially SNPs that have not been associated with an antiviral/anti-retroviral function, or a decrease in such functions) in the context of their RNA editing capabilities. Such potential differences in RNA editing could contribute to the complex differences in immune responses in different individuals, and susceptibility to the development of autoimmune diseases.

Interestingly, A3s are also considered to have an antiviral activity that is deaminase-independent. In double deaminase domain A3s, a mutation in the non-catalytically active domain results in a reduction in antiviral activity [157,158,159,160]. The mechanism of this deaminase independent activity is unknown with some suggesting that RNA binding is important for this process [158], and as previously mentioned, RNA binding inhibits ssDNA deamination [127,128]. In the case of A3A and A3G, where RNA editing has been described, it may be worthy to consider that this ‘deaminase independent’ activity may in fact be DNA deaminase-independent and be a result of the RNA editing of cellular transcripts that by some mechanism increases the intrinsic cellular immunity or RNA editing of the viruses/retroelements. Much more research into these links and the functions of A3 mediated RNA editing is necessary.

Overall, very little is known about C-to-U RNA editing, especially in humans, and much more interest and effort are required to begin to understand its in vivo consequences. It is certainly worthwhile to re-evaluate the A3s’ DNA editing abilities, and to consider other A3s for possible RNA editing functions. Most of the inquiry into the functions of the A3s has involved overexpression studies where proteins are then found in levels that are higher than possible in nature, and activities and targets that may not occur in vivo may be observed. Often, the A3s are studied as a tagged fusion protein; while in some cases, no differences in DNA deamination can be seen, in others, such as human and rhesus monkey A3A a C-terminal tag, greatly reduced anti-viral activity against SHIV [161]. Therefore, this group of proteins should be studied further in their native form under native expression conditions to determine whether humans have evolved a host of different RNA editors. In that context, it is interesting to note that DND1 can also interact with APOBEC3 in mice, with inhibitory effects on miRNA functions [72,78]. In humans A3G appears to be able to interact with HNRNPC1, HNRNPAB, SYNCRIP, and HNRNPR [162,163]; most of these proteins are involved in C-to-U RNA editing and/or related to ACF [164]. Considering the potential role of A3G and A3A in RNA editing [18], it is tempting to speculate that different RBPs, in different tissues, could work as cofactors in bringing the A3s to their specific targets (and consequently, it remains possible that APOBEC1 and A3s contain a conserved region for the interaction with these RBPs). Overall, it remains to be determined if A3A and A3G have RBP interaction partners like ACF and RBM47 (the APOBEC1 interactors) that are a part of the human RNA editing machinery, and what, if anything, determines the specificity of the targets between the proteins and the different cell types.

## 6. Human Diseases Related to C-to-U Editing

Whereas it is clear that C-to-U editing occurs with reasonable frequency, because APOBEC1 expression is thought to be tissue-restricted in humans, most of the disease burden must be associated with A3 RNA editors, and these have not been extensively studied, as noted above. At the same time, whereas all AID/APOBEC family members were originally characterized exclusively, either as DNA mutators or RNA editors, this distinction is not supported either by structural data (all active sites appear near-identical—reviewed in [14,15]) or by cell biological findings (where APOBEC1 can function as a robust DNA mutator [11]). Because most deaminases can edit both DNA and RNA, it is important to keep this in mind as we assess the relevance of RNA editing to specific diseases.

Even though APOBEC1 is not supposed to be expressed outside the human intestinal tract, all genetic dysfunctions recently ascribed to APOBEC1 are within the human brain. *GlyR* editing is important in disease progression of temporal lobe epilepsy (TLE) [165]. Specifically, *GlyR* editing was shown to be increased in the hippocampus of patients with pharmacoresistant temporal lobe epilepsy (TLE) [165]. RNA-edited GlyRs are expressed at the presynaptic terminals of hippocampal neurons [166], and even small differences in editing can induce dysfunction (probably because the low levels of editing identified in bulk represent editing of *GlyR* within specific subsets of neurons) [166,167]. At the same time, it was shown that APOBEC1 is able to edit the *GlyR* mRNA in vitro. Whereas this is only suggestive (as noted above), recent bioinformatic analyses have identified two genetic variants of APOBEC1, 80M and 80I, which are associated with *GlyR* editing levels [168]. Further characterization of patients with intractable TLE (iTLE) for APOBEC1 dimorphism, showed that patients with 80I variant suffer from simple or complex seizures, whereas patients with 80M exhibit neurodegeneration and secondary generalized seizure activity [168]. The I80M polymorphism in human APOBEC1 was reported previously in the small intestine, with no effect on *Apob* RNA editing [169]. The identification of the APOBEC1 80I/M-coding allele as a new genetic risk factor for iTLE patients is the first example of a direct human disease association between APOBEC1 and a tissue other than the small intestine. Similar studies must be carried out with the A3 family, as well as disease entities that APOBEC1 and A3 are involved in.

In the nervous system, C-to-U editing of the transcript encoding tryptophan hydroxylase 2 (TPH2a) can lead to alternative splicing. In the inserted exon, 3b, there is a C-to-U editing event that leads to a recoding mutation (Q129X substitution) and a truncated protein [170]. Editing at this position decreased substantially (by 50% and 30%) in suicide-prone and schizophrenia patients, respectively [170]. To date, the responsible C-to-U editing enzyme remains unknown.

Mutations in the APOBEC1 cofactors are also associated with disease. Mutations in RBM47 have been associated with breast cancer progression, and specifically with an increase in the fitness of certain cancer clones and increased metastatic potential [171]. In addition, expression of RBM47 correlates with a good prognosis in patients with lung, breast, and gastric cancer suggesting a tumor-suppressive role [172]. RBM47 robustly binds around 2500 transcripts, but mRNA editing has not been assessed in these studies [171]. It would be important to assess whether the loss of the RNA editing function in some transcripts due to loss of the editing co-factor is directly responsible for disease progression. In support of this hypothesis, it is important to note that some targets identified as RBM47-bound RNA [171] were also identified as APOBEC1 RNA editing targets or as APOBEC1 RNA interactors, such as β-2 microglobulin (B2m) and interleukin-8 (IL8) [173], respectively.

Polymorphisms in A3 RNA editors could affect human disease progression by influencing immune responses or through changes in RNA editing levels or targets. In autoimmune diseases, systemic lupus erythematosus (SLE) patients often have high levels of circulating type I interferon, and elevated expression of interferon-stimulated genes, which include various A3s [174,175]. These SLE patients can also exhibit increased levels of A-to-I (by adenosine deaminases) and C-to-U RNA editing [176]. This increased level of editing can result in amino acid recoding and the development of potential MHC class I epitopes, which are possible contributors to disease development [176].

The RNA editing of A3A and A3G appears to be more scrupulous than their DNA editing with a more specific sequence signature [177]. Where DNA editing by A3A and A3G appears to be generally non-specific with only the dinucleotide [T/C]C as a preference, RNA editing was more likely to be observed in stem-loop structures, with the target C contained in the loop; the more stable the stem, the higher the levels of editing [177]. The rs172378 C1q synonymous SNP associated with nephritis in SLE patients [178,179], has increased C-to-U editing within three nucleotides from the polymorphism, likely by altering the RNA secondary structure stabilizing a stem-loop [177]. This type of SNP affects how RNA may be targeted by the A3s. While it is unknown whether this change directly contributes to SLE, as with other RNA editing events, it could contribute to the transcript fate and subsequently to the outcome of the protein, and thus contribute to the disease. Likewise, it is possible that other such SNPs, including synonymous ones, could result in changes in RNA editing affecting protein diversity.

A3A and A3G have both also been implicated to play various roles in cancers. It stands to reason that the question of what the additional involvement of RNA editing by these editors in these cancers should be addressed. RNA editing may also account for some of the unclear mechanisms of the involvement of A3G in cancer.

## 7. Conclusions

Research in the field of RNA editing has really taken off, with the advent of NGS technologies [17,18,69], which have demonstrated that C-to-U editing in human is not limited to APOBEC1, but can also be catalyzed by APOBEC3 family enzymes. In that context, it is important to note the discovery of RBM47 as an APOBEC1 co-factor even for *Apob* editing [63]. Additionally, recent work has indicated that co-factors, which are crucial to targeting the editing enzyme to the right transcript, might have distinct transcript preferences. Future work will need to clarify the identity and role of co-factors for APOBEC3 editases, and further clarify the role of co-factors other than ACF and RBM47 for APOBEC1 editing.
